# Phenotypic analysis of mice completely lacking netrin 1

**DOI:** 10.1242/dev.128942

**Published:** 2015-11-01

**Authors:** Andrea R. Yung, Allison M. Nishitani, Lisa V. Goodrich

**Affiliations:** Department of Neurobiology, Harvard Medical School, Boston, MA 02115, USA

**Keywords:** Netrin-1, Axon guidance, Commissural neurons

## Abstract

Netrin 1 (Ntn1) is a multifunctional guidance cue expressed in the ventricular zone and floor plate of the embryonic neural tube. Although Ntn1 is best known for acting as an axon guidance cue through Dcc and neogenin receptors, it is also thought to regulate neuronal survival and blood vessel development through Unc5 family receptors. However, the *Ntn1* gene trap mutant mouse does not display all the phenotypes predicted from *in vitro* assays or analyses of mice lacking predicted receptors. Since the gene trap strain still produces wild-type Ntn1 protein, it is unclear whether the absence of phenotypes reflects the activity of alternative cues or of residual Ntn1. To resolve the full contribution of Ntn1 to development, we generated a null allele of *Ntn1* and re-examined tissues exhibiting phenotypic discrepancies between receptor mutants and *Ntn1* hypomorphs. We found that in *Ntn1* null animals commissural axons rarely cross the midline, resulting in a strongly enhanced phenotype relative to *Ntn1* hypomorphs, which retain many axons with normal trajectories. Thus, low levels of Ntn1 can account for persistent attraction to the midline in hypomorphs. By contrast, *Ntn1* null mice do not show all of the phenotypes reported for Unc5 receptor mutants, indicating that Ntn1 is not necessarily the dominant ligand for Unc5 family members *in vivo* and ruling out primary roles in survival or angiogenesis.

## Abstract (precis)

**Summary:** Complete deletion of the axon guidance cue netrin 1 leads to severe defects in midline crossing of spinal cord axons. However, the mutants did not fully phenocopy loss of the netrin 1 receptor Unc5.

## INTRODUCTION

Netrin 1 (Ntn1) is a secreted molecule of the laminin superfamily (Ishii et al., 1992) that is best known for its role in axon guidance (Serafini et al., 1996), with additional roles in adhesion (Srinivasan et al., 2003; Yebra et al., 2003), angiogenesis (Lu et al., 2004) and survival (Mazelin et al., 2004). To mediate these diverse functions, Ntn1 signals through multiple receptors, including deleted in colorectal cancer (Dcc) (Fazeli et al., 1997), neogenin (Srinivasan et al., 2003), Unc5 family members (Leonardo et al., 1997) and integrins (Yebra et al., 2003). However, as shown by RT-PCR and *in situ* hybridization, the most commonly studied *Ntn1* mutant is a severe hypomorph that does not exhibit all of the phenotypes predicted by *in vitro* assays and phenotypic analyses of Ntn1 receptor mutants (Lu et al., 2004; Serafini et al., 1996; Williams et al., 2006). Another gene trap allele is also available, but is likely to suffer the same issues as the original line (Salminen et al., 2000). Thus, even after 20 years of active research, it is unclear whether the absence of predicted defects is due to redundant cues or residual Ntn1, raising questions about the full contributions of Ntn1 to development *in vivo*.

To resolve lingering questions regarding the broad functions of Ntn1 *in vivo*, we created a null allele of *Ntn1*. Phenotypic analysis of this improved mouse model confirmed a primary role for Ntn1 during midline guidance of commissural axons, but not for all Unc5-mediated effects on repulsion, neuronal survival or blood vessel branching.

## RESULTS AND DISCUSSION

### Wild-type Ntn1 protein persists in *Ntn1^trap/trap^* mice but is absent from *Ntn1*^−/−^ mice

**Fig. 1.**
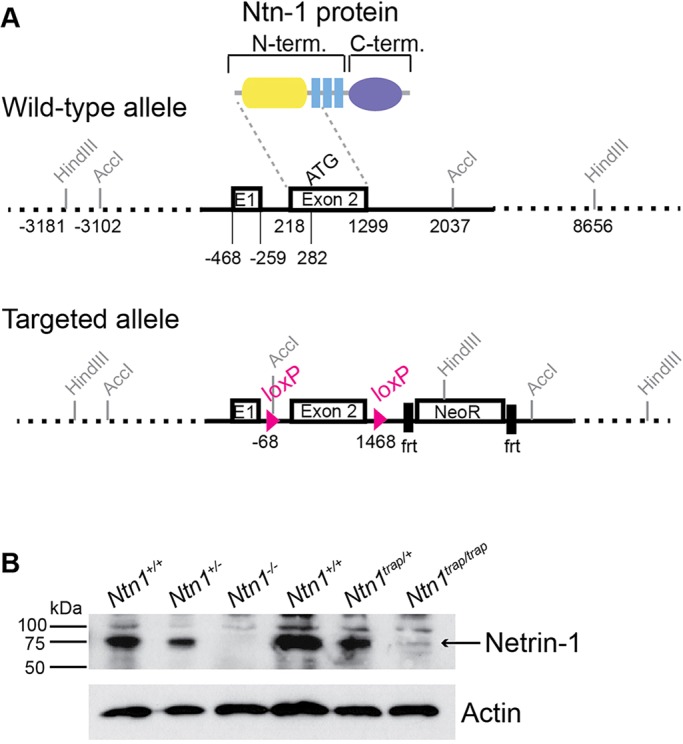
**Generation of the *Ntn1* null mouse.** (A) Map of the wild-type and floxed *Ntn1* loci with GenBank annotations. *loxP* sites flank exon 2; its protein product (yellow, domain VI; blue, domain V) is delineated by dashed lines. (B) Western blots of E11.5 head lysate show residual protein in *Ntn1^trap/trap^* mutants but no detectable protein in the newly generated *Ntn1^−/−^* mutants. Loading controls (actin) were obtained from a shorter exposure of the same gel.

Western blot analysis of E11.5 head lysate with an antibody targeted to Ntn1 domain VI (Bin et al., 2013) revealed that residual wild-type protein (∼75 kDa) persists in *Ntn1* gene trap (*Ntn1^trap/trap^*) animals (*n*=2), confirming that this allele is hypomorphic. To generate a null mouse, we inserted *loxP* sites around the second exon of *Ntn1*, which encodes the start codon and most of the N-terminus and, when fused to Fc, is sufficient for axon outgrowth *in vitro* (Keino-Masu et al., 1996; Lim and Wadsworth, 2002). We crossed this floxed *Ntn1* allele to the germline-specific Cre line *EIIa^Cre^* to delete exon 2 from subsequent generations (Fig. 1A). In contrast to gene trap mutants, no Ntn1 protein was detected in *Ntn1^−/−^* animals (Fig. 1B; *n*=4), which die neonatally without any gross malformations: E18.5 null embryos were present in Mendelian ratios (33/121 embryos), but no *Ntn1^−/−^* pups (out of 51) were observed at P5.

### Ntn1 is the major cue for midline attraction

As a chemoattractant, Ntn1 acts through Dcc and neogenin (Xu et al., 2014) to promote the growth and guidance of dorsally located commissural neurons toward the ventral floor plate (Serafini et al., 1996). However, although many commissural axons misproject to the ventricular zone and the motor columns in *Ntn1* hypomorphs, a subset of axons still orient toward and reach the floor plate. These observations led many groups to look for additional floor plate-derived cues, resulting in the discovery that VEGF (Ruiz de Almodovar et al., 2011) and sonic hedgehog (Shh) (Charron et al., 2003) also function as chemoattractants. Unfortunately, the persistence of Ntn1 in *Ntn1^trap/trap^* mice makes it difficult to distinguish the contributions of these cues from those of Ntn1 during nervous system wiring.

**Fig. 2.**
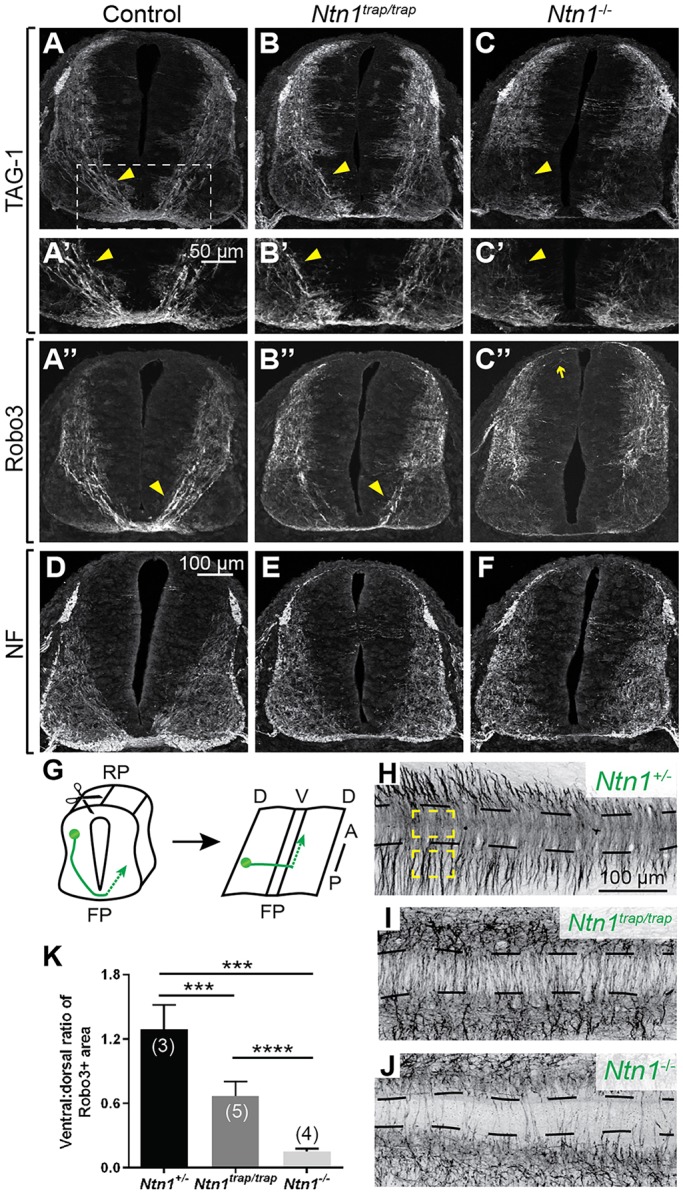
**The *Ntn1^trap/trap^* commissural phenotype is enhanced in *Ntn1^−/−^* mutants.** (A-C″) Low (A-C,A″-C″) and high (A′-C′) magnification views of E11.5 spinal cord sections stained for TAG-1 and Robo3 reveal that fewer commissural axons (arrowheads) cross the midline in *Ntn1^−/−^* animals (C) compared with controls (A) and *Ntn1^trap/trap^* hypomorphs (B), with some axons projecting dorsally (arrow). (D-F) Neurofilament (NF) stains show grossly normal organization of the spinal cord in E11.5 null mutants. (G-J) Robo3 staining of open-book preparations of E11.5 spinal cords (G) show that fewer axons cross the midline (dashed lines) in *Ntn1^trap/trap^* animals (I) compared with controls (H). This phenotype is more severe in *Ntn1^−/−^* animals (J). (K) Quantification of the midline crossing phenotypes illustrated in H-J. Yellow boxes in H indicate the dorsal and ventral areas quantified. RP, roof plate; FP, floor plate; D, dorsal; V, ventral. ****P<0.0001, ***P<0.001; Mann–Whitney test. Error bars indicate s.d.

To assess the extent of Ntn1-independent commissural axon guidance, we stained E11.5 *Ntn1^−/−^* spinal cord sections for the commissural markers TAG-1 (Cntn2 – Mouse Genome Informatics) and Robo3 (Sabatier et al., 2004; Serafini et al., 1996). In wild-type embryos, fasciculated axons travel along the lateral edge of the neural tube, turn ventromedially at the motor columns, and cross the floor plate (Fig. 2A; *n*=3). Since no differences were observed between wild-type and heterozygous animals, both genotypes were used as controls. In *Ntn1^trap/trap^* mutants, some axons still arrive at the floor plate with normal trajectories (Fig. 2B; *n*=2), consistent with Serafini et al. (1996)*.* By contrast, *Ntn1^−/−^* mutants display defasciculated TAG-1^+^ and Robo3^+^ axons that project towards the ventricular zone into the motor columns or even dorsally (Fig. 2C; *n*=6). Very few axons appear to cross the midline. However, the gross organization of the spinal cord was normal, with sensory and motor axons growing through the expected entry and exit points (Fig. 2D-F; *n*=4).

To quantify the extent of midline crossing in *Ntn1^−/−^* animals, we stained for Robo3^+^ commissural axons at the floor plate in open-book preparations of E11.5 spinal cords and calculated the ratio of ventral to adjacent dorsal areas covered by Robo3^+^ axons in a ∼500 µm segment of the cervical-thoracic spinal cord (Fig. 2G-K). Both *Ntn1^trap/trap^* and *Ntn1^−/−^* mutants displayed highly disorganized commissural axons that were often oriented away from the midline. However, the degree of crossing was significantly decreased in *Ntn1^−/−^* embryos (*n*=4) compared with *Ntn1^trap/trap^* (*n*=5) and *Ntn1*^+/−^ (*n*=3) animals (Fig. 2K).

**Fig. 3.**
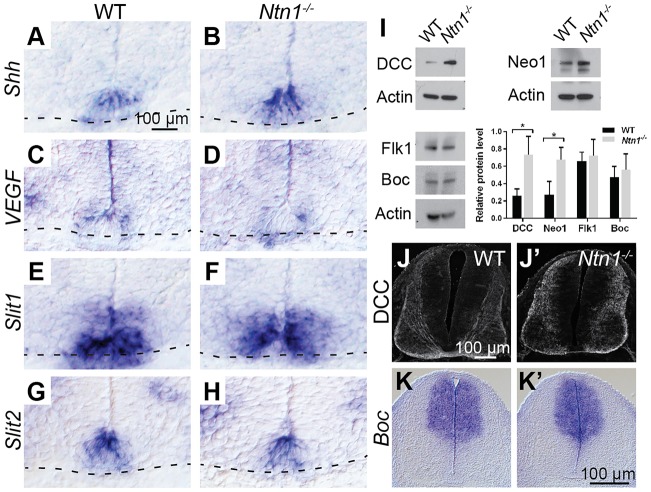
***Ntn1^−/−^* mutants maintain expression of other guidance cues and their receptors.** (A-H) *In situ* hybridization of E11.5 spinal cord sections; dashed lines indicate the ventral edge. *Shh* (A,B), *Vegf* (C,D), *Slit1* (E,F) and *Slit2* (G,H) are expressed at the floor plate of mutant animals, as in wild-type (WT) controls. (I) Quantified western blots for Dcc, neogenin, Flk1 and Boc show similar, or upregulated, levels of these receptors in wild-type and null animals. *P<0.05; Student's *t*-test. Error bars indicate s.d. (J-K′) Immunostaining (J,J′) and *in situ* hybridization (K,K′) confirm that Dcc and *Boc* expression is preserved in E11.5 mutant spinal cords.

To investigate whether the enhanced strength of the commissural phenotype observed in *Ntn1^−/−^* animals is secondary to changes in the availability of other chemoattractants, we examined the expression of other floor plate-derived cues and their receptors in *Ntn1* mutants. We found that floor plate identity is preserved, as revealed by *in situ* hybridization of *Shh*, a floor plate marker and short-range chemoattractant for commissural axons (Charron et al., 2003), the chemoattractant *Vegf* (*Vegfa*) (Ruiz de Almodovar et al., 2011) and the chemorepellants *Slit1* and *Slit2* (Long et al., 2004) (Fig. 3A-H; *n*=3 null embryos). Moreover, a combination of western blotting (*n*=3 E11.5 heads per genotype), immunostaining (*n*=3 animals per genotype) and *in situ* hybridization (*n*=2 animals per genotype) confirmed that the Shh receptor Boc (Okada et al., 2006), the VEGF receptor Flk1 (Kdr – Mouse Genome Informatics) (Ruiz de Almodovar et al., 2011), and Dcc and neogenin are still present (Fig. 3I-K), so the enhanced phenotype should not be due to a lack of responsiveness. On the contrary, Dcc and neogenin levels were significantly increased in *Ntn1* mutants (Fig. 3I), similar to observations from an independent null strain (Bin et al., 2015), implying that Ntn1 regulates the availability of its own receptors.

Together, these data suggest that, although other cues and their receptors are present, they may not compensate for the absence of Ntn1. Low levels of Ntn1 in the hypomorph are therefore sufficient to attract many commissural axons to the midline, consistent with reports that fewer than five Ntn1 molecules can induce growth (Pinato et al., 2012). In the absence of Ntn1, some axons still grow ventrally, likely by chance, at which point attractants such as VEGF and Shh may guide them across the midline. Indeed, Shh can induce turning but not outgrowth, and mice lacking Shh or VEGF activity show relatively subtle guidance phenotypes (Charron et al., 2003; Ruiz de Almodovar et al., 2011). Thus, whereas Ntn1 guides over long distances to establish the overall pathway, other cues act at close range to fine-tune axon trajectories within this framework.

### Trochlear nerve projections remain intact in *Ntn1*^−/−^ mice

The striking contrast in the severity of the *Ntn1^trap/trap^* versus *Ntn1^−/−^* commissural phenotype highlights the potent attractive capabilities of Ntn1 even at reduced levels. This raised the possibility that low levels of Ntn1 remaining in hypomorphs might have obscured other guidance defects *in vivo*. In addition to acting as an attractant, Ntn1 has been proposed to repel trochlear motor neurons, which reside in the ventral hindbrain and project axons dorsally, away from the floor plate. However, although Ntn1 repels trochlear axons from hindbrain explants *in vitro* and loss of the Ntn1 receptor *Unc5c* causes guidance defects in the trochlear nerve *in vivo*, no defects in the trochlear nerve have been observed in *Ntn1^trap/trap^* animals (Burgess et al., 2006; Colamarino and Tessier-Lavigne, 1995; Serafini et al., 1996; Varela-Echavarría et al., 1997).

**Fig. 4.**
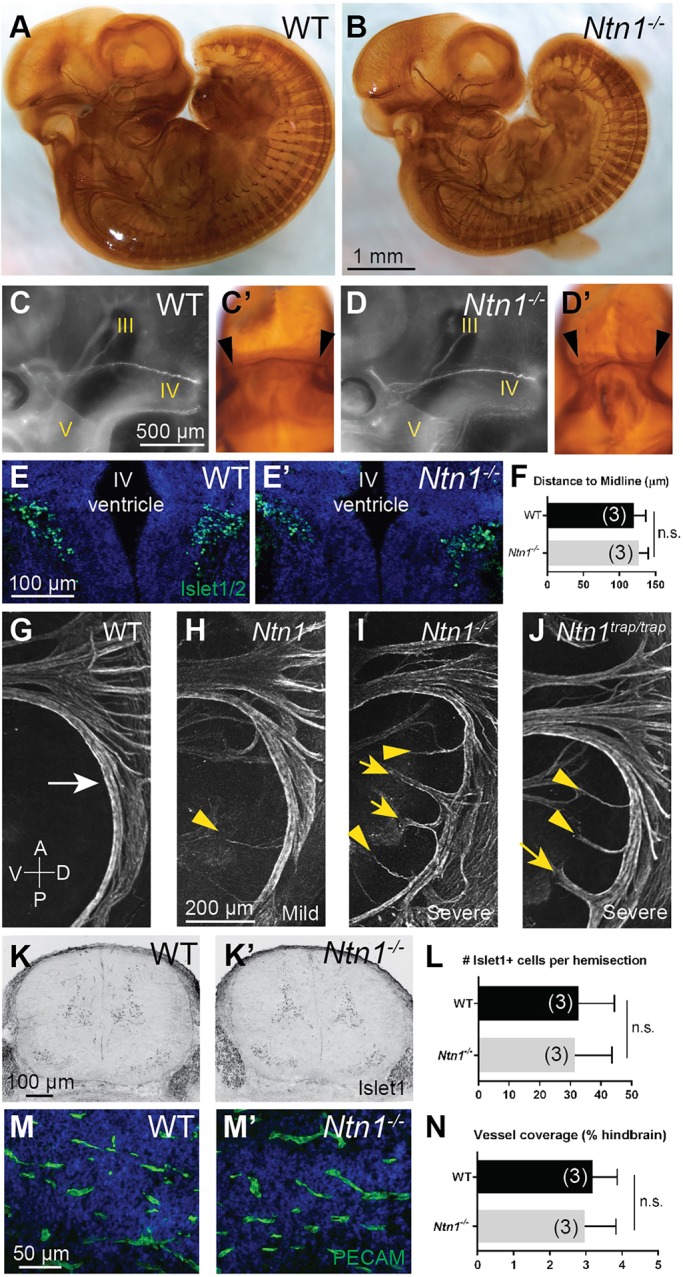
***Ntn1^−/−^* mutants do not display many known Unc5 receptor mutant phenotypes.** (A-D′) Colorimetric and fluorescent wholemount neurofilament stains show comparable sensory and motor projections in wild-type (A) and *Ntn1* null (B) E11.5 embryos, including normal trochlear (IV) nerve trajectories (C,D) with an intact dorsal decussation (C′,D′; arrowheads indicate trochlear nerve on either side). Cranial nerves III, IV and V are indicated. (E-F) Islet1/2-positive trochlear nuclei retain their normal position relative to the midline in mutant E12.5 coronal hindbrain sections, as quantified in F. (G-J) SACMN axons normally form a smooth, hook-shaped nerve (white arrow), but both null (H,I) and hypomorphic (J) mutants show variable defects, ranging from a few axons (yellow arrowheads) to whole bundles of axons (yellow arrows) wandering away from the nerve at many positions. (K-L) Islet1 immunostains show no change in the number of motor neurons in the spinal cord of E13.5 wild-type and null animals, as quantified in L. (M-N) Immunostaining for PECAM in E12.5 hindbrain sections show no change in blood vessel coverage, as quantified in N. n.s., not significant (F, *P*=0.474; L, *P*=0.445; N, *P=*0.480; Mann–Whitney test). Error bars indicate s.d.

To determine if residual Ntn1 obscured a role in trochlear pathfinding, we visualized peripheral axons in E11.5 *Ntn1^−/−^* embryos by performing wholemount neurofilament staining. The overall pattern of sensory and motor projections appeared unchanged (Fig. 4A,B), and we detected no qualitative differences in the presence, trajectory or dorsal decussation of the trochlear nerve in null mutants (Fig. 4C,D; *n*=4 wild-type, *n*=8 *Ntn1^−/−^* embryos). Additionally, the position of the trochlear nuclei relative to the midline, as shown by Islet1/2 immunostaining at E12.5, was similar in controls and mutants (Fig. 4E,F; *n*=3 animals per genotype). These data suggest that Ntn1 does not act as the major repulsive cue for trochlear axons and that other ligands are responsible for Unc5c-dependent growth and guidance of the trochlear nerve.

### Absence of other Unc5-mediated phenotypes in *Ntn1^−/−^* mice

Given the discordance between the *Ntn1**^−/−^*** and *Unc5c**^−/−^*** trochlear nerve phenotypes, we investigated whether other Unc5-mediated activities might also be independent of Ntn1. We first examined projections from spinal accessory motor neurons (SACMNs), which showed variable defects in dorsal migration and guidance in *Ntn1* hypomorphs and in mice mutant for Dcc or Unc5c (Dillon et al., 2005, 2007). SACMNs are ventrally located in the cervical spinal cord and extend axons dorsally along the lateral edge of the spinal cord before exiting and turning rostrally to form a longitudinal, hook-shaped nerve (Fig. 4G). All mutants (*n*=13 nerves) displayed spinal accessory nerve abnormalities in at least one side of the body, but the phenotype remained variable. In mildly affected nerves a few axons wander ventrally (Fig. 4H), whereas in more severe cases entire segments of the nerve branch ventrally (Fig. 4I), similar to what is observed in severely affected gene trap mutants (Fig. 4J). Thus, residual Ntn1 does not explain the partial penetrance of the SACMN guidance defect in *Ntn1* hypomorphs, indicating that closer comparison of Dcc- and Unc5c-dependent SACMN populations is warranted.

Intriguingly, in *Unc5a^−/−^* mutants, these same neurons are less susceptible to cell death, hinting that Ntn1 might also function as a survival cue (Williams et al., 2006). However, *in vivo* evidence for this model is lacking, as SACMN number was unchanged in *Ntn1^trap/trap^* embryos, perhaps due to its hypomorphic nature. To resolve this issue we stained E13.5 cervical spinal cord sections and observed no significant difference in the number of Islet1-positive SACMNs per hemisection in serial sections between wild-type (*n*=8) and null (*n*=8) embryos (Fig. 4K,L), suggesting that Unc5a-mediated survival of SACMNs occurs through another ligand. Consistent with this interpretation, cell death also appeared unaffected in a different *Ntn1^−/−^* strain (Bin et al., 2015).

Given the mismatch in receptor and ligand neural phenotypes *in vivo*, we sought to clarify whether Unc5 receptors also act independently of Ntn1 during blood vessel development. Although there are no obvious vascular malformations in *Ntn1* hypomorphs, *Unc5b* mutants die at E12.5 with excessive blood vessel branching in numerous regions, including the hindbrain (Lu et al., 2004). Despite complete loss of Ntn1, there were no obvious differences in blood vessel coverage in wild-type (*n*=3) versus null (*n*=3) animals (Fig. 4M,N), as assessed by staining E12.5 hindbrain sections for the vascular marker PECAM (Pecam1– Mouse Genome Informatics). Together with the lack of trochlear and SACMN survival phenotypes, these data imply that other ligands might be more important than Ntn1 for the activation of Unc5 receptors in many contexts.

A growing body of literature has shown that members of the fibronectin and leucine-rich transmembrane protein family mediate repulsion through Unc5 receptors in multiple systems (Yamagishi et al., 2011). Similarly, Draxin, an axon guidance molecule expressed in the dorsal neural tube, acts on the same neurons that respond to Ntn1 and can bind to both Dcc and Unc5 family members (Islam et al., 2009). It is tempting to speculate that these molecules could contribute to trochlear nerve guidance, although compensation by other Netrin family members might also play a role. As increasing numbers of ligands for Ntn1 receptors are identified, this has led to an appreciation of the diversity of molecular cues available to direct brain wiring and invites broader consideration about how receptors integrate signals from multiple ligands during circuit formation and other developmental processes.

It is somewhat surprising that complete loss of *Ntn1* did not uncover any obvious novel phenotypes, given its broad expression and demonstrated potency. Evidence for additional roles might emerge on different genetic backgrounds, since lethality occurs earlier in another *Ntn1^−/−^* strain (Bin et al., 2015). Indeed, the enhanced commissural phenotype highlights the need to re-examine other tissues that show phenotypes in receptor mutants but not *Ntn1^trap/trap^* animals. The *Ntn1* floxed allele described in this study provides an ideal tool to study other developmental functions within and beyond the nervous system, particularly in postnatal and adult animals, as illustrated by a recent study of sympathetic arterial innervation (Brunet et al., 2014).

## MATERIALS AND METHODS

### Generation of a floxed *Ntn1* allele

Using the NM_008744 *Ntn1* cDNA as a reference, a linker sequence containing a *loxP* site was inserted upstream of exon 2 in a 2.85 kb 5′ arm of homology (−1412 to +1442 bp), which was cloned into the 4600c vector (gift of Richard Palmiter, University of Washington), with a 3.45 kb 3′ arm (+1423 to +4873 bp) inserted via synthetic *Xho*I and *Cla*I sites downstream of a second *loxP* sequence and *frt*-flanked neomycin cassette. After *Asc*I linearization, the targeting construct was electroporated into J1 ESCs (derived from the 129S4/SvJae strain), and selected under G418. Recombinant clones were identified by Southern blot using external 5′ and 3′ probes. The neomycin resistance cassette was removed by crossing *Ntn1^floxed-neo/+^* mice to a global *FLPe* driver [JAX, B6;SJL-Tg(ACTFLPe)9205Dym/J] (Rodríguez et al., 2000), yielding floxed *Ntn1* mice (*Ntn1^fl/+^*) as confirmed by real-time PCR (Transnetyx).

A null allele (*Ntn1^+/−^*) was created by crossing *Ntn1^fl/+^* animals to a germline *Cre* driver [JAX, B6.FVB-Tg(EIIa-cre)C5379Lmgd/J] (Lakso et al., 1996). Genotyping was performed with primers spanning the deleted exon (AMN363, 5′-CAGGTGGCAAGAGAAAAGGA-3′; and AMN437, 5′-TCCGTTTGGATCTGGGATTA-3′) and with primers inside the deleted exon (AMN357, 5′-CTCAATAACCCGCACAACCT-3′; and AMN358, 5′-CTCCGAGTCGTCTTCGTTCT-3′): wild type, 468 bp; *Ntn1^−/−^*, 432 bp. *Ntn1^+/−^* animals used in this study were backcrossed to C57BL/6 animals for three to five generations.

### Animals

The *Ntn1* gene trap line was reported previously (Serafini et al., 1996) and has been backcrossed to C57BL/6 animals for more than ten generations. Noon on the day of the plug was considered embryonic day (E) 0.5. All animal work was conducted in compliance with protocols approved by the Institutional Animal Care and Use Committee at Harvard Medical School.

### Immunoblotting

E11.5 heads were lysed in 50 mM Tris pH 7.4, 150 mM NaCl, 1% NP-40, 0.5% sodium deoxycholate, 0.1% SDS, 1× Pefabloc SC PLUS protease inhibitor (Roche). Primary antibodies used include goat anti-Boc (1:1000; R&D, AF2385), goat anti-Dcc (1:1000; Santa Cruz, SC-6535), rabbit anti-Flk1 (1:2000; Cell Signaling; as described in Gu et al., 2003), goat anti-neogenin 1 (1:1000; R&D, AF1079) and rat anti-Ntn1 (1:500; R&D, AF1109). Each blot was performed twice using independent lysates.

### *In situ* hybridization

*In situ* hybridization was performed on 20 µm frozen sections as described (Lu et al., 2011).

### Immunochemistry

For sections, embryos were fixed in 4% paraformaldehyde (PFA) in PBS at 4°C overnight, cryoprotected in sucrose, embedded in Neg50 (VWR, #84000-156) and cryosectioned at 12 or 20 µm. Primary antibodies used were mouse anti-Islet1/2 (1:100; DSHB), anti-neurofilament (1:1000; DSHB), goat anti-Robo3 (1:100; AF3076) and goat anti-TAG-1 (1:1000; R&D, AF4439).

For wholemounts, embryos were fixed in 4% PFA in PBS at 4°C overnight, dehydrated in methanol, incubated in Dent's bleach and fixative, and rehydrated in PBS. Samples were blocked overnight (20% DMSO, 5% normal goat serum, 0.3% Triton X-100 and 0.025% sodium azide in PBS), incubated with mouse anti-neurofilament for 5 days at 4°C, washed in blocking solution, and incubated with secondary antibody at room temperature for 2 days. Embryos were cleared in BABB [1:2 benzyl alcohol (Sigma B-1042): benzyl benzoate (Sigma B-6630)]. Staining of open-book spinal cord samples followed the same protocol, but after fixation the tissue was placed directly in blocking solution.

Spinal cord sections and wholemount embryos were imaged on an Olympus FV1000 confocal microscope using 10×, 0.40 NA or 20×, 0.75 NA dry objectives and with optimal step sizes in the *z-*axis (1.16 µm steps for 20× and 4.27 µm steps for 10×). Open-book wholemounts were imaged on a Leica SP8 X confocal microscope with a 20×, 0.70 NA objective. Quantification was performed using ImageJ (NIH), where coverage denotes the percentage of a standardized area covered by either Robo3^+^ or PECAM^+^ pixels; two independent measurements were taken per open-book. To measure the distance between the trochlear nucleus and the midline, a straight line was drawn from the center of the nucleus to the IVth ventricle. All statistical analyses were performed with Prism 4 (GraphPad software); all data are presented as mean±s.d.
